# Application of three-dimensional reconstruction combined with dial positioning in small pulmonary nodules surgery

**DOI:** 10.1186/s13019-021-01642-4

**Published:** 2021-09-08

**Authors:** Long Zhao, Wenyu Yang, Ruofeng Hong, Jianbin Fei

**Affiliations:** 1 Cardiovascular Surgery Department, Hwa Mei Hospital, University of Chinese Academy of Sciences, Ningbo City, China; 2 Ningbo Institute of Life and Health Industry, University of Chinese Academy of Sciences, Ningbo City, China

**Keywords:** 3-D reconstruction, Dial positioning, VATS, Pulmonary nodules

## Abstract

**Background:**

With the popularization of HRCT and VATS, the incidence of early stage lung cancer is increasing recent years. About 63% of small pulmonary nodules can not be accurately identified in VATS. We use 3-D reconstruction combined with dial positioning to analyze its accuracy and impact on patients undergoing VATS in our hospital.

**Method:**

All patients underwent HRCT scanning and 3-D reconstruction preoperatively to determine the scope of surgery. The precise positional relationship between the nodule and the nearest rib must be recorded. Locate the plane of pulmonary nodule on CT, rotate the plane to make the affected side upwards, take the highest point of pleura as 12 o'clock on the dial, record the corresponding point of the nodule meticulously, mark the pulmonary nodule on the skin of the patient. A 18G indwelling needle was used to puncture through the marker into the visceral pleura. Electrocautery mark was made on the bleeding point of the lung surface. Then wedge resection or segmental resection was made.

**Materials and result:**

From September 2019 to December 2020, 74 patients underwent VATS pulmonary nodule resection in our institute, with an average age of (56.4 ± 11.7) years old. A total of 83 nodules were resected in 74 patients, 23 nodules received segmentectomy and 60 nodules received wedge resection with 16 benign nodules and 67 malignant nodules. The distance between the nodules and pleura was (0–25) mm, with an average of (8.0 ± 3.9) mm. The target nodules were found in all patients, the positioning accuracy was 97.6%. All patients were satisfied with the positioning method, and there was no scar left at the skin puncture point after operation.

**Conclusion:**

3-D reconstruction combined with dial positioning method can reduce patients' anxiety preoperatively, avoid various complications, reduce hospitalization expenses, and has an acceptable accuracy and short learning curve, which can be further promoted and applied in clinic.

## Background

With the popularization of high resolution computed tomography (HRCT) and video assisted thoracoscopic surgery (VATS), the incidence of early stage lung cancer is increasing recent years [1]. When the nodule is less than 10 mm in the size or more than 5 mm away from the pleura, about 63% of them can not be accurately identified in VATS [2]. How to locate and resect them accurately is the key to treatment [3, 4]. In this study, we use three-dimensional (3-D) reconstruction technique combined with dial positioning to analyze its accuracy and impact on patients undergoing VATS in our hospital.

## Materials

From September 2019 to December 2020, 74 patients (28 males and 46 females) underwent VATS pulmonary nodule resection in our institute, with an average age of (56.4 ± 11.7) years old ranging from 26 to 85 years old. Inclusion criteria: (1) patients’ nodules were deemed to have VATS; (2) the depth of the nodule from the pleura was more than 5 mm, or the nodule was not visible thoracoscopically; (3) only wedge or segmental resection was reckoned preoperatively. Exclusion criteria: (1) the depth of the nodule from the pleura was more than 30 mm or nodules located near the spine or covered by the scapula or at the apex of the lung; (2) lobectomy was reckoned; (3) extensive adhesion of thoracic cavity was found during operation. This study has been approved by the medical ethics committee of the hospital.

## Method

All patients underwent HRCT scanning and 3-D reconstruction with mimics 21.0 software preoperatively to locate the pulmonary nodule and determine the scope of surgery.

**Fig. 1:**
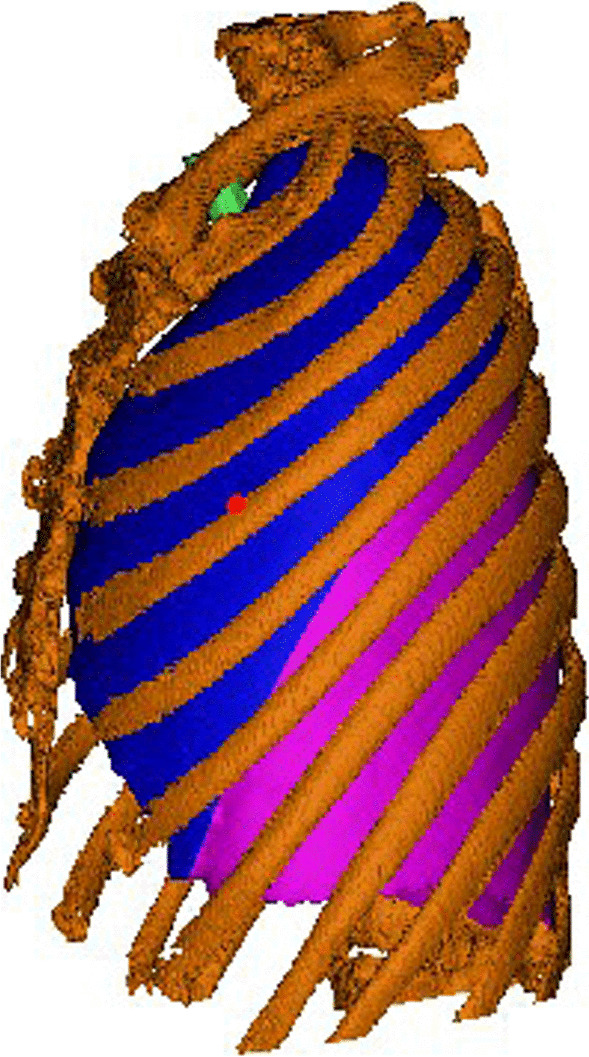
The nodule is at the upper margin of the left fourth rib

1.1 All the patient’s HRCT images were downloaded and saved as DICOM (Digital Imaging and Communications in Medicine) format from the PACS (Picture Archiving and Communication Systems) in our hospital, then a 3-D image of lung was reconstructed by mimics 21.0 software. After marking the nodule and distinguishing the segments, ribs were added on the 3-D image to facilitate the localization of the nodule. The precise positional relationship between the nodule and the nearest rib must be recorded (Fig. 1).

**Fig. 2:**
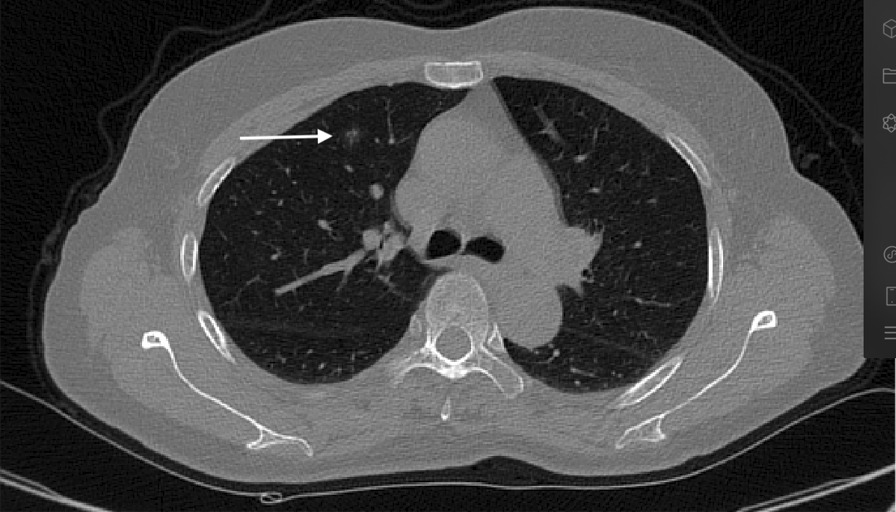
The nodule is on the anterior segment of the right upper lung lobe

**Fig. 3:**
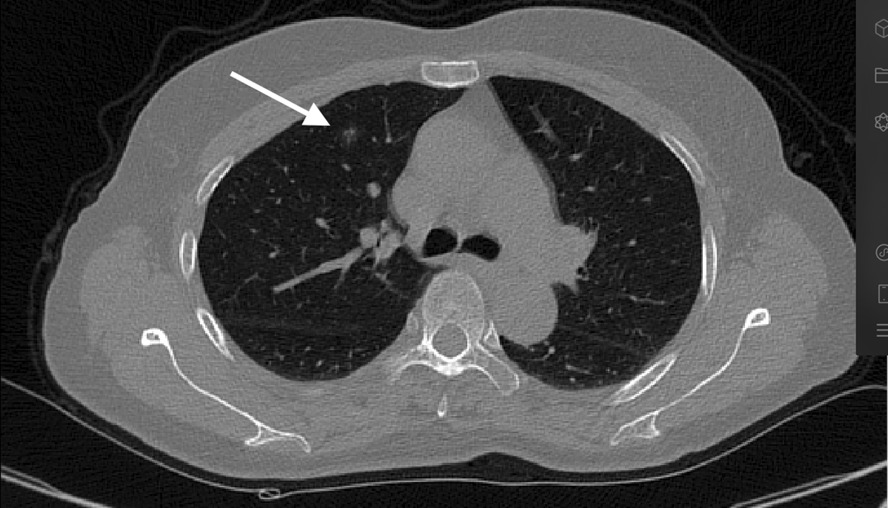
Rotate the plane, so that the affected side is upward

**Fig. 4:**
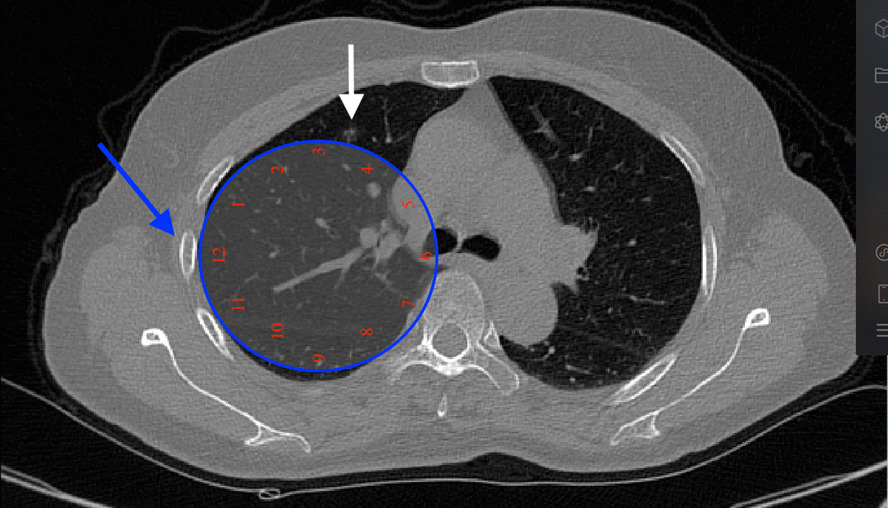
The 4th rib is at the 12 o’clock and the nodule is at the 3:30 of this plane. White arrows indicate the nodule, blue arrow indicates the 4th rib

1.2 Locate the plane of pulmonary nodule on horizontal CT (Fig. 2), record the rib or intercostal space of the plane which is not always explicit, it is necessary to translate the plane, for example: the plane is 5 layers below the inferior margin of the 4th rib at the midclavicular line. Then rotate the plane so that the affected side is upward (Fig. 3), take the highest point of pleura as 12 o'clock on the dial, record the corresponding point of the nodule meticulously (Fig. 4).

**Fig. 5:**
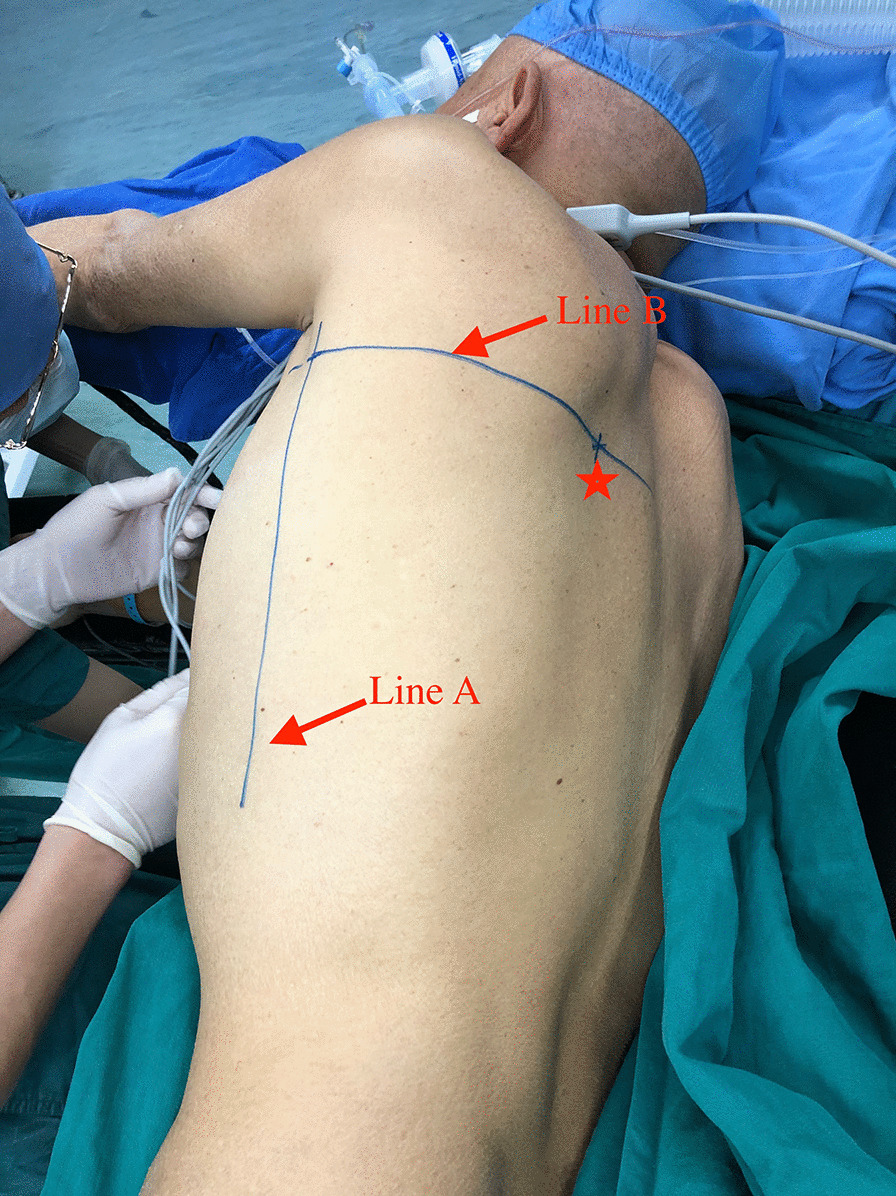
Line A, through the highest points of the ribs; Line B, the plane in which the nodule located; ★, the marker of the nodule

After anesthesia, patients were positioned laterally with the affected side facing up, draw a line (Line A) through the highest points of the ribs. Mark the rib or intercostal space on the line according to the 3-D reconstruction and CT positioning, draw another line (Line B) perpendicularly through the rib or intercostal space, mark the pulmonary nodule on the second line according to the location of the dial (Fig. 5).

Routine thoracoscopic exploration was carried out after the lung slightly collapsed. A 18G indwelling needle was used to puncture vertically to the body surface from the positioning point into the subpleural. Inflated the lung and pushed the needle in to break the visceral pleura, and then selective ventilation was performed. Electrocautery mark was made on the bleeding point of the lung surface. Then wedge resection or segmental resection was made.

Observation index: (1) The transverse distance between the positioning point and the pulmonary nodule was recorded, which no more than 2.0 cm was regarded as accurate positioning, while more than 2.0 cm was regarded as positioning deviation. The number of patients with accurate positioning and deviation cases were recorded, and the positioning accuracy was calculated.

(2) 9The postoperative complications were recorded.

### Statistical analysis

Data are presented as mean ± standard deviation with range for continuous variables, unless otherwise indicated. Frequency and percentage are listed for categorical variables.

## Results

In 2 patients, squeezing the surrounding lung tissue was needed to find the bleeding point on the visceral pleura after the punctuation. In other patients, electrocoagulation marks were made after the lung collapse directly.

The target nodules were found in all patients, 2 of them were more than 2 cm away from the electrocoagulation point, which means positioning deviation, and the others were less than 2 cm. The positioning accuracy was 97.6%.

**Fig. 6:**
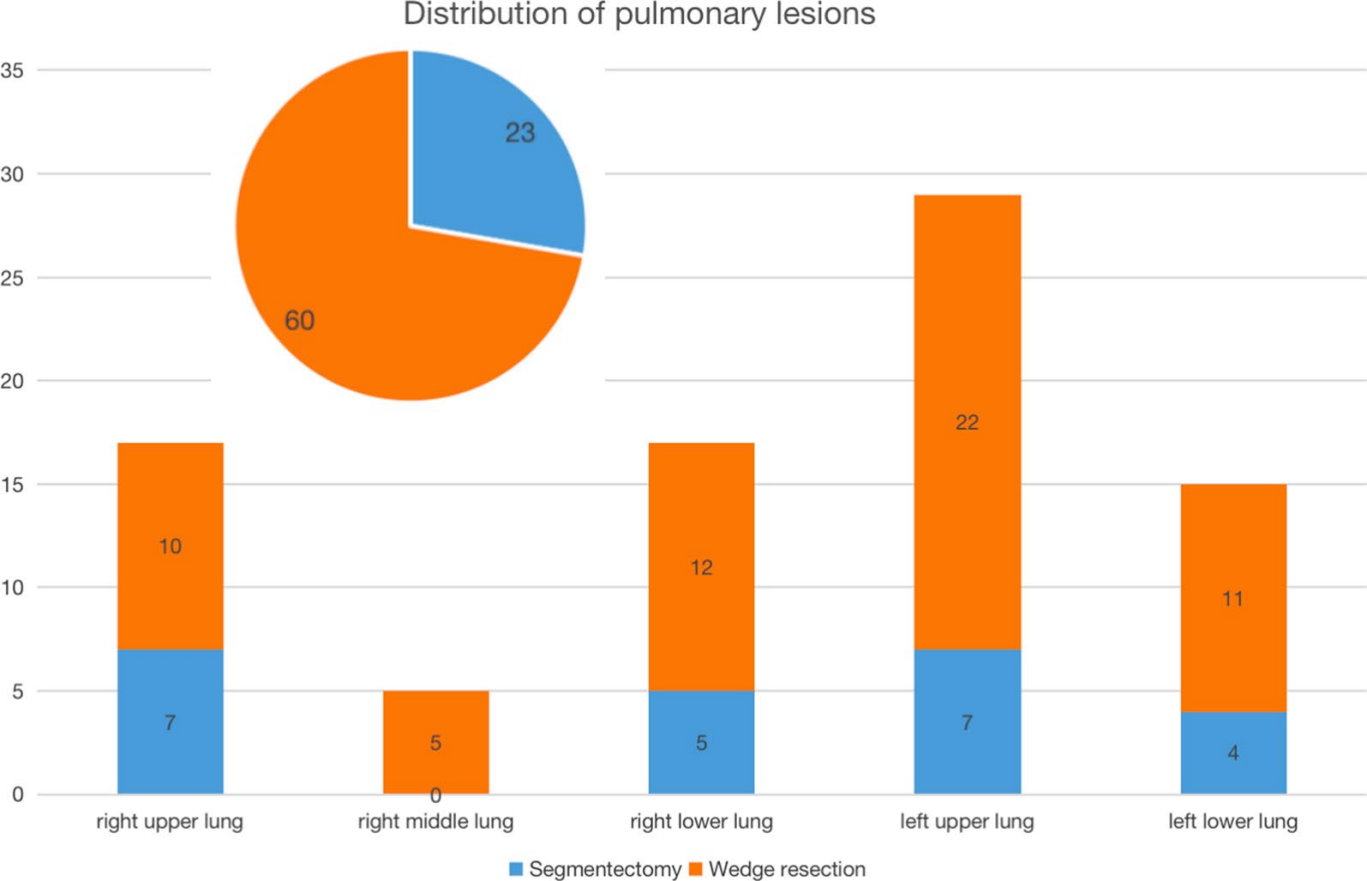
83 nodules were resected, 17 nodules in the right upper lung (20.5%), 5 nodules in the right middle lung (6%), 17 nodules in the right lower lung (20.5%), 29 nodules in the left upper lung (34.9%) and 15 nodules in the left lower lung (18.1%)

A total of 83 nodules were resected in 74 patients (Fig. 6). 23 nodules received segmentectomy and 60 nodules received wedge resection. There were 16 benign nodules and 67 malignant nodules (Table 1). The distance between the nodules and pleura marker was (0–25) mm, with an average of (8.0 ± 3.9) mm (Table 2).

**Table 1:** There were 16 benign nodules and 67 malignant nodules

	n
Total nodules	83
Benign lesions	
Granulomatous nodule	11
Tuberculosis	1
Inflammatory nodule	4
Malignant lesions	
Invasive adenocarcinoma	25
Minimally invasive adenocarcinoma	30
Adenocarcinoma in situ	7
Squamous cell carcinoma	3
Metastatic lesions	1
Small cell carcinoma	1

**Table 2:** Distance between the nodules and pleura marker

	Right upper lung	Right middle lung	Right lower lung	Left upper lung	Left lower lung	Total
Distance (mm)						
0–10	12	5	13	24	13	67
11–20	4	0	4	5	1	14
21–30	1	0	0	0	1	2

All patients have not experienced anxiety with the positioning method preoperatively, there was no scar left at the skin puncture point after operation and no bleeding, pneumothorax, tumor implantation and metastasis happened. 3-D reconstructions were performed by researchers and the 18G needles were free, compared with traditional positioning method, patients paid no extra fee.

## Discussion

At present, the incidence and mortality of lung cancer are still in the first place in malignant tumors [5]. VATS has been fully proved to be effective in removing lesions due to its high safety and minimal invasion [6]. However, due to the limitations of VATS, it is difficult to accurately locate small non-superficial pulmonary nodules, which leads to the occurrence of residual nodules after surgery and jeopardize the prognosis [7]. Therefore, how to locate small pulmonary nodules accurately, efficiently and noninvasively becomes one of the keys to successful operation. Traditional localization of pulmonary nodules are mainly carried out by preoperative puncture, using hookwire, spring coil, or injecting with methylene blue, etc. Due to the shortcomings of falling off, displacement, bleeding, pneumothorax, tumor implantation and metastasis, or dye dispersion [8–11], a novel positioning method was needed to ease the anxiety of patients who are already nervous before operation and increase the efficiency of operation.

The dial positioning method used in this study is a real-time positioning after anesthesia and thoracotomy, which can effectively avoid hemothorax, pneumothorax, etc. Electrocoagulation marking after puncturing the pleura decrease the risk of tumor planting and diffusion. Positioning was made in the operation room with no need of CT assistance, patients get no exposure of radiation and have less anxiety before surgery compared with those underwent traditional localization. Bleeding point was not found in the visceral pleura of 2 patients after puncturing, which we considered was related to subpleural carbon deposition and pulmonary fibrosis. By slightly squeezing the surrounding lung tissue, bleeding points and bubbles could be observed and electrocoagulation was made. Deviation happened on two patients, the reason we thought may be the non-vertically puncturation to the body surface. Some studies have shown that the accuracy of hookwire puncture positioning can reach 97% [12], the result of our study is comparable. In this study, the satisfaction of patients was investigated and analyzed. The results showed that the patients had no discomfort to the intraoperative puncture and were satisfied with the positioning results. However, this localization method has some limitations. For the deeper located nodules, only the plane can be marked, accurately location could be challengeable. During the operation, pulling the lung tissue may cause inaccurate positioning which may affect the result of wedge resection, while segmental resection is not affected. For obesity and nodules located near the apex of lung or covered by the scapula, the needle may not be able to enter the thoracic cavity due to the angle or length, so it can not be applied. In addition, the sample size of this study is small, which leads to the decrease of the reference value of the results. In the future, more research samples will be included to further confirm the safety and effectiveness of intraoperative body surface localization of pulmonary nodules.

In conclusion, 3-D reconstruction combined with dial positioning method can reduce patients' anxiety preoperatively, avoid various complications caused by puncturing, reduce hospitalization expenses, and has an acceptable accuracy, which can be further promoted and applied in clinic.

## Data Availability

Yes.

## Abbreviations

HRCT: Resolution computed tomography
VATS: Video assisted thoracoscopic surgery
3-D: Three-dimensional
DICOM: Digital Imaging and Communications in Medicine
PACS: Picture Archiving and Communication Systems
